# Experimental study of the effects of diazepam on vasospasm in a subarachnoid rat model through pathological and biochemical analysis

**DOI:** 10.1038/s41598-025-08325-3

**Published:** 2025-08-09

**Authors:** Eyüp Çetin, Cumaali Demirtaş, Cansu Sönmez, Murat Yücel, Eray Metin Güler, Sarper Kocaoğlu, Hakan Beyaztaş, Emine Demir

**Affiliations:** 1 Neurosurgery Clinic, Haydarpaşa Training and Research Hospital, Health Sciences University, Istanbul, Turkey; 2 Veterinary Clinic, Hamidiye Experimental Animals Laboratory, Health Sciences University, Istanbul, Turkey; 3 Pathology Clinic, Haydarpaşa Numune Hospital, Health Sciences University, Istanbul, Turkey; 4 Faculty of Medicine, Neurosurgery Clinic, Yalova University, Yalova, Turkey; 5 Department of Biochemistry, Hamidiye Medical Faculty, Health Sciences University, Istanbul, Turkey; 6 Department of Medical Biochemistry, University of Health Sciences, Haydarpaşa Numune Health Application and Research Center, Istanbul, Turkey; 7 Neurosurgery Clinic, Training and Research Hospital, Istanbul, Turkey; 8 Department of Medical Biochemistry, Hamidiye Institute of Health Sciences, University of Health Sciences Turkey, Istanbul, Turkey; 9 Department of Neurosurgery, Üsküdar State Hospital, Istanbul, Turkey

**Keywords:** Subarachnoid hemorrhage (SAH), Cerebral vasospasm, Diazepam, Oxidative stress, Experimental models of disease, Molecular neuroscience, Neuro-vascular interactions

## Abstract

Subarachnoid hemorrhage (SAH), characterized by bleeding in the subarachnoid space, is associated with high morbidity and mortality, primarily due to cerebral vasospasm. Recent studies suggest oxidative stress and inflammation play crucial roles in vasospasm pathogenesis. This study investigates the effects of diazepam, a benzodiazepine with vasodilatory properties, in a rat SAH model. Three groups of female Sprague Dawley rats were analyzed: a control group, an SAH-induced group without treatment, and an SAH-induced group treated with 3 mg/kg of diazepam. Our findings revealed SAH significantly increased Total Oxidant Status (TOS), Oxidative Stress Index (OSI), and inflammatory markers (IL-1β, IL-6, TNF-α) in both tissue and serum samples. Diazepam treatment mitigated these effects, showing reduced TOS, OSI, and cytokine levels compared to the untreated SAH group. Additionally, diazepam helped maintain thiol-disulfide balance, with higher Total Thiol and Native Thiol levels, indicating a protective effect against oxidative damage. Histopathological examination revealed significant vasospasm and inflammatory infiltration in the SAH group, which was partially alleviated in the diazepam-treated group. Diazepam may serve as an adjunct therapy in SAH management by modulating oxidative stress and inflammation, potentially alleviating vasospasm and related ischemic injuries.

## Introduction

The incidence of subarachnoid hemorrhage (SAH) varies among ethnic groups. The average incidence rate of aneurysmal SAH is 6–7 per 100,000 people^1^. SAH is most commonly observed in individuals aged 60 and older. It has a mortality rate of 30% and a morbidity rate of 40–50%^2^. Cerebral vasospasm is the reversible narrowing of cerebral arteries that typically develops between days 3–7 after SAH and gradually resolves after the 14th day^3,4^. Vasospasm is a primary factor contributing to morbidity and mortality associated with SAH, yet despite extensive clinical, laboratory, and experimental research over the years, certain aspects of vasospasm remain unclear^5^. Experimental animal studies have proposed numerous theories regarding the pathogenesis of SAH, leading to the conclusion that vasospasm has a multifactorial pathogenesis that cannot be explained by a single mechanism^6^. Some of these studies suggest that vascular wall changes resulting in vascular pathologies involve cellular immunity and immune mechanisms, which, combined with inflammation, contribute to vasospasm^7^. This inflammation-induced vasospasm is primarily driven by inflammatory cytokines, including tumor necrosis factor-alpha (TNF-alpha), interleukin-1beta (IL-1β), and interleukin-6 (IL-6)^8^.

Arterial blood enters the subarachnoid space, cisterns, and typically the ventricular system at the initial rupture, leading to a higher-pressure system. As intracranial pressure (ICP) rises, cerebral perfusion pressure (CPP) decreases (CPP = MAP (mean arterial pressure)-ICP). Under normal physiological conditions, a decrease in CPP results in a limited reduction in cerebral blood flow (CBF) until autoregulatory dysfunction and failure occur. However, in cases of sudden and significant ICP increase, as seen in high-grade SAH, CBF markedly decreases, causing vasodilation of distal cerebral arterioles and a corresponding rise in arterial blood pressure, thereby increasing CBF. This increase in ICP may continue until complete cessation of CBF, resulting in what is known as “transient global cerebral ischemia"^9^.

Angiographic (asymptomatic) vasospasm is detected in 70–99% of patients by the 7th day post-hemorrhage. All SAH patients are administered oral nimodipine (class I), which improves neurological outcomes but does not reduce the risk of vasospasm^10^.

Traditional treatments have focused on vascular resistance, flow viscosity, and blood pressure within the cerebral vasculature. The “Triple H” therapy involves hypervolemia, hemodilution, and hypertension to enhance CBF, though it is associated with complications such as pulmonary edema, myocardial infarction, pneumonia, hyponatremia, and nosocomial infections^11^. Recommendations in guidelines from Japan, Europe, Korea, the USA, and Croatia differ. Prophylactically, oral nimodipine, fasudil (only in Japan), and euvolemia are recommended, while induced hypertension post-DCI (Delayed Cerebral Ischemia) onset is generally suggested in newer guidelines, though evidence supporting induced hypertension remains weak^12^.

Since the 1950 s, diazepam has been one of the most successful and effective drugs of the psychopharmacological revolution, widely used for treating insomnia, anxiety, epilepsy, pain, depression, muscle spasms, convulsions, alcohol withdrawal, and anesthesia induction. Its wide therapeutic range, rapid onset, reliable efficacy, high bioavailability, low toxicity, and low cost have made diazepam one of the most widely used drugs in history^13^. Despite its popularity as a classic benzodiazepine, diazepam has side effects, including drug tolerance, dependence, misuse, memory loss, withdrawal symptoms, falls, and even death^14^.

To achieve vasodilatory effects, micromolar concentrations of benzodiazepines are required. These effects are thought to be modulated through the interaction of benzodiazepines with micromolar benzodiazepine binding sites functionally linked to voltage-gated calcium channels. In addition, benzodiazepines may induce vascular relaxation through mechanisms such as the inhibition of purine uptake^15^. This study investigated the effects of diazepam, a benzodiazepine with vasodilator properties, on experimental SAH in rats.

## Materials and methods

### Animals

Female Sprague-Dawley rats weighing between 200 and 250 g were used in this study. All animal models were handled according to Directive 2010/63/EU of the European Parliament and the Council on the protection of animals used for scientific purposes, as well as relevant European Environment Agency guidelines. The experimental procedures were reviewed and approved by the Health Sciences University Hamidiye Animal Experiments Local Ethics Committee. This study is reported in accordance with the ARRIVE guidelines (PLoS Biol 8(6), e1000412, 2010) for animal research.

The use of female rats was intentional to ensure hormonal consistency and minimize aggression, which can be more pronounced in males. Additionally, to control for potential confounding effects due to the oestrous cycle, all animals were screened for oestrous phase via vaginal smear cytology. Only rats in the dioestrus phase were included in the study. The dioestrus phase was selected because it is associated with relatively stable and low oestrogen levels, which helps reduce hormonal variability that could influence vascular reactivity and inflammatory responses. By synchronizing the oestrous cycle across all animals, we aimed to eliminate any potential confounding effects of fluctuating oestrogen levels on the experimental outcomes.

### Experiment groups

Three groups of 6 rats each were formed:

Group 1 was the control group without SAH induction,

Group 2 had SAH induced but received no diazepam, and.

In Group 3, SAH was induced and 3 mg/kg diazepam (Diazem ampule, Deva İlaç A.Ş., Istanbul) was administered intraperitoneally (injected one hour and 24 h after SAH).

The dose of diazepam (3 mg/kg) was chosen based on previous rodent studies showing its effectiveness in reducing oxidative stress without causing sedative toxicity. This dose was effective in regulating oxidative and inflammatory responses while minimizing possible sedative side effects^16^.

### SAH model induction

**Fig. 1:**
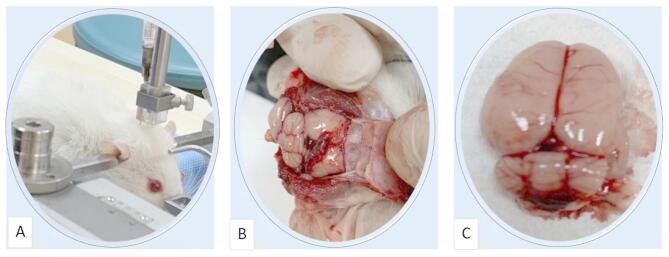
Creation of the SAH model. **A** Taking 0.2 ml of CSF from the cisterna magna of the rat placed in the stereotaxic device and injecting the same volume of non-heparinized arterial blood. **B** Cisterna magna. **C** Subarachnoid hemorrhage.

To establish the SAH model, rats were fasted for 12 h. Anesthesia was induced by intraperitoneal injection of 50 mg/kg Ketamine Hydrochloride (Ketalar bottle, Eczacıbaşı İlaç, İstanbul, under license from Parke-Davis) and 10 mg/kg Xylazine (Rhompun injection bottle, Bayer Türk Kimya, İstanbul). Animals were placed in a stereotaxic apparatus. The entire surgical procedure, including positioning and blood injection into the subarachnoid space, is illustrated in Fig. 1. 0.2 ml of CSF was collected from the cisterna magna using a 30G needle. The same volume of non-heparinized blood taken from the femoral artery was injected into the cisterna magna. After the surgical procedure, rats were allowed to breathe spontaneously, kept at normal room temperature (20–22 °C), and provided with standard rat chow and sufficient water.

### Sacrifice and sample collection

48 h after SAH was induced, all animals underwent cervical dislocation under general anesthesia and 3 ml of intracardiac blood was collected into biochemistry tubes containing clot activator and gel. Blood samples were centrifuged at 3000 g for 10 min to separate the serum and stored at −80 °C until analysis. After euthanasia, rats underwent frontoparietal occipital craniectomy and intracranial structures extending to the foramen magnum were carefully removed and stored in 10% formaldehyde solution.

### Histopathological examination

**Fig. 2:**
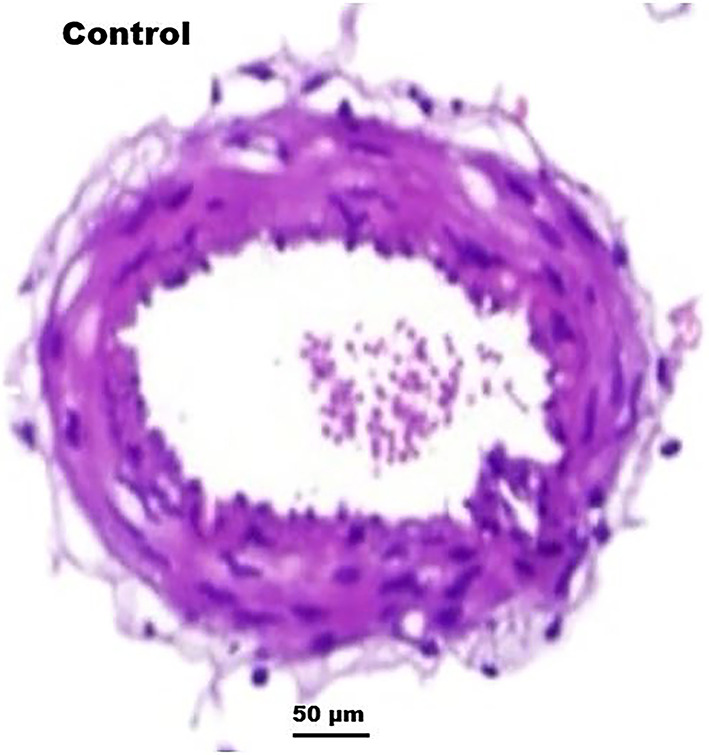
Normal basilar artery structure in control group. Histological appearance of the basilar artery in control group rats, demonstrating normal lumen diameter, uniform wall thickness, and absence of inflammation. (Note: Hematoxylin & Eosin staining; magnification, 50x).

Euthanized rats were subjected to frontoparietal occipital craniectomy, and intracranial structures extending to the foramen magnum were preserved intact in 10% buffered formaldehyde solution. Samples were then processed and embedded in paraffin blocks. Sections of 5 μm thickness were cut from the paraffin blocks, stained with hematoxylin-eosin, and examined under a light microscope by two blinded observers, demonstrating normal basilar artery morphology with preserved lumen and uniform wall thickness in control animals (Fig. 2). Measurements were taken for basilar artery lumen area and basilar artery wall thickness. We used the Argenit Easypath digital scanning system to quantitatively measure bacillary artery diameter.

### Examination of biochemical parameters

Biochemical parameters were measured in serum and in the supernatant obtained from tissue homogenates. All tissue-based parameters were normalized based on protein levels.

#### Biochemical quantification

The levels of IL-1β (IL-1β, BTLab-E0119Ra, Shanghai, China), IL-6 (IL-6, BTLab-E0135Ra, Shanghai, China), and TNF-α (TNF-α, BTLab-E0764Ra, Shanghai, China) in samples were determined using commercially available Enzyme-Linked ImmunoSorbent Assay (ELISA) kits (Shanghai, China). Measurements were performed according to the manufacturer’s instructions, and absorbance values were recorded at 450 nm using a BioTek Synergy™ HTX Multi-Mode Reader. The accuracy of the ELISA assays was validated using control samples with known biomarker concentrations, and intra- and inter-assay coefficient of variation (CV) was maintained below 10%.

Oxidative stress was assessed by quantifying TAS and TOS using commercially available kits (RelAssay, Mega Tıp, Gaziantep, Turkey). OSI was calculated as the ratio of TOS to TAS, providing a comprehensive measure of oxidative balance. In serum samples, TAS values were expressed in mmol ascorbic acid equivalents per liter (mmol AA Eq./L), while TOS levels were reported in µM hydrogen peroxide equivalents per liter (µM H₂O₂ Eq./L). For tissue samples, oxidative stress parameters were normalized to total protein content to account for variations in sample concentration.

The redox balance within samples was evaluated by measuring Total Thiol (TT) and Native Thiol (NT) levels using commercially available kits (RelAssay, Mega Tıp, Gaziantep, Turkey). Disulfide (DIS) levels were calculated using the formula:

TT and NT concentrations were expressed in micromolar (µM), while the relative ratios NT/TT, DIS/TT, and DIS/NT were presented as percentages (%). In tissue samples, these values were normalized to total protein content to ensure accurate comparisons across samples.

#### Tissue homogenization

Frontal tissue samples were first allowed to equilibrate to room temperature and then precisely weighed. Homogenization was carried out using a QIAGEN TissueLyser LT system (Hilden, Germany) in 1× phosphate-buffered saline (PBS; 0.1 M, pH 7.4). Following homogenization, the samples were centrifuged at 13,000 rpm for 10 min using a Beckman Coulter Allegra^®^ X-30 centrifuge (USA). The supernatants obtained after centrifugation were collected and stored for subsequent biochemical analyses.

To quantify total protein content, the Coomassie Plus Protein Assay Kit (Thermo Fisher Scientific, Massachusetts, USA) was employed. Absorbance readings were taken at 595 nm using a BioTek Synergy™ HTX Flash Multimode Reader. Protein concentrations were determined by referencing a standard curve generated from serial dilutions of known protein standards.

### Statistical methodology

Descriptive statistics for the data included the mean, standard deviation (SD), median, minimum, maximum, frequency, and ratio values. The Kolmogorov-Smirnov and Shapiro-Wilk tests were used to assess normality, while the Kruskal-Wallis and Mann-Whitney U tests were applied for non-normally distributed quantitative independent variables. All statistical analyses were performed using SPSS version 27.0.

## Results

### Group distribution

The study population was equally divided into three groups: Control (*n* = 6), Diazepam-treated (*n* = 6), and SAH-induced (*n* = 6), each comprising 33.3% of the total sample. This balanced group distribution ensured valid statistical comparisons across different conditions.

### Baseline measurements and descriptive statistics

The descriptive statistics of biochemical and histopathological parameters in both tissue and serum samples are presented in Table 1. The basilar artery diameter in tissue ranged from 88.7 to 226.8 micrometers, with a mean ± SD of 140.1 ± 36.8 micrometers. The arterial wall thickness varied from 20.1 to 62.1 micrometers, with a mean ± SD of 38.9 ± 11.2 micrometers. Serum TOS had a mean of 7.2 ± 2.2, whereas TAS had a mean of 0.7 ± 0.3. These baseline values provide a reference for understanding the oxidative stress and antioxidant levels across different groups.

**Table 1:** Descriptive statistics of oxidative stress markers, inflammatory cytokines, and vascular parameters in tissue and serum samples.

		Min–Max	Median	Mean ± SD
*Tissue*				
Basilar artery diameter		88.7–226.8	136.8	140.1 ± 36.8
Arterial wall thickness		20.1–62.1	34.9	38.9 ± 11.2
Total oxidant status		2.3–9.8	4.0	4.8 ± 2.2
Total antioxidant status		0.4–0.9	0.7	0.7 ± 0.1
Oxidative stress index		3.3–15.1	5.2	7.7 ± 4.7
Interleukin–1beta		4.4–10.8	7.2	7.6 ± 1.7
Interleukin-6		1.1–5.2	2.9	3.1 ± 1.2
Tumor necrosis factor alpha		37.3–127.8	74.5	80.7 ± 28.5
Hypoxia inducible factor 1 alpha		0.3–1.0	0.6	0.7 ± 0.2
Cytokeratin 18-M65		125.7–367.8	194.8	228.7 ± 88.2
*Serum*				
Total oxidant status		3.9–10.5	7.0	7.2 ± 2.2
Total antioxidant status		0.3–1.2	0.8	0.7 ± 0.3
Oxidative stress index		3.5–27.2	9.0	12.2 ± 7.7
Interleukin–1beta		7.3–27.7	11.9	15.0 ± 7.0
Interleukin–6		7.4–29.9	15.2	17.4 ± 8.5
Tumor necrosis factor alpha		91.8–382.1	211.7	226.2 ± 93.2
Hypoxia inducible factor 1 alpha		1.5–5.8	2.3	2.9 ± 1.5
Cytokeratin 18–M65		88.1–427.7	223.2	241.3 ± 114.9
Total thiol		254.8–569.1	399.4	391.8 ± 97.1
Native thiol		74.2–393.4	219.8	222.9 ± 116.4
Disulfide		40.9–148.9	82.2	84.4 ± 29.2
% Native thiol/total thiol		21.7–81.4	56.6	53.7 ± 19.8
% Disulfide/total thiol		9.3–39.1	21.7	23.1 ± 9.9
% Disulfide/native thiol		11.4–180.2	38.6	59.9 ± 50.9
Group distribution	Control group		6	33.3%
	Diazepam group		6	33.3%
	SAH group		6	33.3%

### Oxidative stress and antioxidant markers

Oxidative stress indicators differed significantly among the groups in both tissue and serum samples. In tissue samples (Table 2), TOS was significantly elevated in the SAH group (7.6 ± 1.2, *p* = 0.002) compared to the Control (3.0 ± 0.2) and Diazepam (3.9 ± 0.9) groups. Similarly, TAS was significantly lower in the SAH group (0.55 ± 0.08, *p* = 0.002) than in the Control (0.80 ± 0.07) and Diazepam (0.72 ± 0.08) groups. The OSI, a combined measure of oxidative and antioxidant balance, was highest in the SAH group (13.9 ± 1.1, *p* = 0.001), significantly exceeding the values observed in the Control (3.8 ± 0.3) and Diazepam (5.3 ± 1.2) groups.

**Table 2:** Comparison of tissue oxidative stress markers, inflammatory cytokines, and vascular parameters among control, diazepam, and SAH groups.

		^1^Control Group (*n*=6)	^2^Diazepam Group (*n*=6)	^3^SAH Group (*n*=6)	*p*	
*Tissue*						
Basilar artery diameter	Mean ± SD	153.7 ± 46.4	139.9 ± 40.4	126.6 ± 19.5	0.431	^K^
	Median	143.2	143.1	132.0		
Arterial wall thickness	Mean ± SD	42.2 ± 11.2	35.5 ± 11.5	38.9 ± 11.8	0.554	^K^
	Median	37.2	33.4	36.2		
Total oxidant status	Mean ± SD	3.0 ± 0.2	3.9 ± 0.9	7.6 ± 1.2	***0.002***	^K^
	Median	3.0^3^	4.0^3^	7.3		
Total antioxidant status	Mean ± SD	0.80 ± 0.07	0.72 ± 0.08	0.55 ± 0.08	***0.002***	^K^
	Median	0.80	0.72	0.55^1,2^		
Oxidative stress index	Mean ± SD	3.8 ± 0.3	5.3 ± 1.2	13.9 ± 1.1	***0.001***	^K^
	Median	3.8^2,3^	5.2^3^	14.0		
Interleukin-1beta	Mean ± SD	6.0 ± 1.0	7.4 ± 0.9	9.5 ± 0.8	***0.002***	^K^
	Median	6.4^2,3^	7.2^3^	9.6		
Interleukin-6	Mean ± SD	1.7 ± 0.3	3.0 ± 0.3	4.6 ± 0.3	***0.001***	^K^
	Median	1.8^2,3^	2.9^3^	4.5		
Tumor necrosis factor alpha	Mean ± SD	54.3 ± 11.5	71.4 ± 10.3	116.3 ± 7.6	***0.001***	^K^
	Median	57.5^2,3^	74.5^3^	113.0		
Hypoxia inducible factor 1 alpha	Mean ± SD	0.45 ± 0.09	0.59 ± 0.10	0.94 ± 0.08	***0.001***	^K^
	Median	0.45^2,3^	0.58^3^	0.96		
Cytokeratin 18-M65	Mean ± SD	151.4 ± 27.6	191.3 ± 24.1	343.4 ± 21.7	***0.001***	^K^
	Median	143.8^2,3^	186.8^3^	347.6		

^K^Kruskal–Wallis (Mann–Whitney u test).

^1^Significant difference from Control Group *p*<0.05.

^2^Significant difference from Diazepam Group *p*<0.05.

^3^Significant difference from SAH Group *p*<0.05.

Serum samples (Table 3) showed a similar trend. TOS was significantly increased in the SAH group (9.7 ± 0.6, *p* = 0.003) relative to the Control (5.7 ± 1.8) and Diazepam (6.1 ± 0.9) groups. Serum TAS levels were lowest in SAH rats (0.45 ± 0.07, *p* = 0.006) compared to the Control (1.01 ± 0.19) and Diazepam (0.75 ± 0.23) groups. The OSI values were also significantly higher in SAH (21.8 ± 3.5, *p* = 0.002) compared to the Control (5.9 ± 2.4) and Diazepam (9.0 ± 3.3). These findings indicate a significant increase in oxidative stress and a marked reduction in antioxidant defenses in SAH rats.

**Table 3:** Comparison of serum oxidative stress markers, inflammatory cytokines, and thiol/disulfide homeostasis among control, diazepam, and SAH groups.

		^1^Control Group (*n*=6)	^2^Diazepam Group (*n*=6)	^3^SAH Group (*n*=6)	*p*	
*Serum*						
Total oxidant status	Mean ± SD	5.7 ± 1.8	6.1 ± 0.9	9.7 ± 0.6	***0.003***	^K^
	Median	5.5^3^	6.1^3^	9.7		
Total antioxidant status	Mean ± SD	1.01 ± 0.19	0.75 ± 0.23	0.45 ± 0.07	***0.006***	^K^
	Median	1.09	0.84	0.45^1^		
Oxidative stress index	Mean ± SD	5.9 ± 2.4	9.0 ± 3.3	21.8 ± 3.5	***0.002***	^K^
	Median	4.9^3^	7.8^3^	22.0		
Interleukin-1beta	Mean ± SD	9.0 ± 1.8	13.4 ± 4.6	22.5 ± 5.6	***0.002***	^K^
	Median	8.1^3^	11.6^3^	25.1		
Interleukin-6	Mean ± SD	9.9 ± 5.1	14.6 ± 3.6	27.6 ± 1.8	***0.001***	^K^
	Median	7.7^2,3^	13.9^3^	27.3		
Tumor necrosis factor alpha	Mean ± SD	124.1 ± 27.8	223.9 ± 32.1	330.4 ± 46.8	***0.001***	^K^
	Median	120.9^2,3^	211.7^3^	343.1		
Hypoxia inducible factor 1 alpha	Mean ± SD	1.6 ± 0.1	2.4 ± 0.4	4.8 ± 0.7	***0.001***	^K^
	Median	1.6^2,3^	2.3^3^	4.7		
Cytokeratin 18-M65	Mean ± SD	128.2 ± 21.0	209.5 ± 33.2	386.1 ± 40.2	***0.001***	^K^
	Median	135.9^2,3^	223.2^3^	385.5		
Total thiol	Mean ± SD	481.2 ± 49.0	413.8 ± 52.4	280.3 ± 42.5	***0.002***	^K^
	Median	476.1	399.4^1^	258.6^1,2^		
Native thiol	Mean ± SD	359.9 ± 18.2	223.8 ± 12.3	85.0 ± 16.3	***0.001***	^K^
	Median	357.2	219.8^1^	49.2^1,2^		
Disulfide	Mean ± SD	60.6 ± 19.3	95.0 ± 29.7	97.6 ± 24.9	***0.030***	^K^
	Median	58.7^2,3^	86.6	90.5		
% Native thiol/total thiol	Mean ± SD	75.2 ± 5.7	54.9 ± 8.0	31.0 ± 8.2	***0.001***	^K^
	Median	75.4	56.6^1^	29.9^1,2^		
% Disulfide/total thiol	Mean ± SD	12.4 ± 2.8	22.6 ± 4.0	34.5 ± 4.1	***0.001***	^K^
	Median	12.3^2,3^	21.7^3^	35.1		
%Disulfide/native thiol	Mean ± SD	16.8 ± 5.0	43.0 ± 15.3	119.9 ± 40.9	***0.001***	^K^
	Median	16.4^2,3^	38.6^3^	117.8		

^K^Kruskal–Wallis (Mann–Whitney u test).

^1^Significant difference from Control Group *p*<0.05.

^2^Significant difference from Diazepam Group *p*<0.05.

^3^Significant difference from SAH Group *p*<0.05.

### Inflammatory markers

Markers of inflammation, including interleukins and TNF-α, were significantly altered across the groups in both tissue and serum samples. In tissue samples (Table 2), (IL-1β was significantly increased in the SAH group (9.5 ± 0.8, *p* = 0.002) compared to the Control (6.0 ± 1.0) and Diazepam (7.4 ± 0.9) groups. IL-6 levels were also highest in the SAH group (4.6 ± 0.3, *p* = 0.001), exceeding those observed in the Control (1.7 ± 0.3) and Diazepam (3.0 ± 0.3) groups. TNF-α, a key pro-inflammatory cytokine, was significantly elevated in the SAH group (116.3 ± 7.6, *p* = 0.001) compared to the Control (54.3 ± 11.5) and Diazepam (71.4 ± 10.3) groups. Hypoxia Inducible Factor 1 Alpha (HIF-1α), a biomarker of cellular response to hypoxia, was significantly higher in SAH (0.94 ± 0.08, *p* = 0.001) than in the Control (0.45 ± 0.09) and Diazepam (0.59 ± 0.10) groups.

Serum samples (Table 3) revealed similar patterns, with IL-1β significantly elevated in SAH rats (22.5 ± 5.6, *p* = 0.002) compared to the Control (9.0 ± 1.8) and Diazepam (13.4 ± 4.6) groups. IL-6 levels were highest in the SAH group (27.6 ± 1.8, *p* = 0.001), surpassing those in the Control (9.9 ± 5.1) and Diazepam (14.6 ± 3.6) groups. TNF-α was significantly elevated in the SAH group (330.4 ± 46.8, *p* = 0.001), compared to the Control (124.1 ± 27.8) and Diazepam (223.9 ± 32.1) groups. Similarly, serum HIF-1α levels were significantly increased in SAH rats (4.8 ± 0.7, *p* = 0.001) compared to the Control (1.6 ± 0.1) and Diazepam (2.4 ± 0.4) groups. These results indicate a strong inflammatory response in the SAH group, while Diazepam showed partial protective effects in reducing inflammation.

### Cytokeratin 18-M65 and cellular damage markers

Cytokeratin 18-M65, a biomarker of cellular apoptosis and necrosis, was significantly increased in SAH rats. In tissue samples (Table 2), CK-18 M65 levels were significantly higher in the SAH group (343.4 ± 21.7, *p* = 0.001) compared to the Control (151.4 ± 27.6) and Diazepam (191.3 ± 24.1) groups. Similarly, serum CK-18 M65 levels (Table 3) were significantly elevated in SAH rats (386.1 ± 40.2, *p* = 0.001) compared to the Control (128.2 ± 21.0) and Diazepam (209.5 ± 33.2). These findings suggest extensive cellular damage in SAH-induced rats.

### Thiol-disulfide homeostasis

Thiol-disulfide homeostasis, a key marker of oxidative stress and protein oxidation, was significantly altered in SAH rats. Total Thiol and Native Thiol levels were significantly lower in SAH rats compared to the Control and Diazepam groups. In contrast, Disulfide levels were significantly higher in the SAH group, confirming a more oxidized redox state. These findings further support the hypothesis that SAH is associated with increased oxidative stress and protein oxidation.

The findings indicate that SAH induces significant oxidative stress, inflammation, and cellular damage, as evidenced by increased levels of TOS, OSI, pro-inflammatory cytokines, and CK-18 M65 in both tissue and serum samples. TAS levels were significantly reduced, indicating a weakened antioxidant defense system. Thiol-disulfide balance also shifted towards a more oxidized state, further confirming the increased oxidative burden in SAH-induced rats. Diazepam exhibited some protective effects by reducing oxidative stress and inflammatory markers compared to the SAH group, but these effects were not sufficient to restore normal levels. These results highlight the need for further investigation into potential therapeutic strategies to mitigate SAH-induced oxidative damage.

## Discussion

This study investigated the effects of diazepam on oxidative stress, inflammatory responses, and thiol-disulfide balance in a SAH rat model. Our findings suggest that diazepam may have a partially protective role against the severe oxidative and inflammatory damage typically associated with SAH, aligning with emerging perspectives on the importance of anti-inflammatory and antioxidant therapies in SAH management. SAH is associated with high morbidity and mortality due to complications such as cerebral vasospasm, a condition where cerebral arteries constrict following SAH, often resulting in delayed ischemia and further brain injury^1,2^. Despite advancements in understanding SAH pathophysiology, the specific mechanisms underlying vasospasm and delayed ischemic injury remain incompletely understood^3,5,17^.

Our findings align with prior studies showing oxidative stress as a central factor in SAH-related vascular injury^7,9,18^. The elevation of TOS and OSI in the SAH group (Tables 2 and 3) underscores the significant oxidative damage following SAH. Elevated ROS levels following blood degradation in the subarachnoid space disrupt cellular and mitochondrial structures, which impairs endothelial function and increases the risk of cerebral vasospasm^7^. The diazepam-treated group demonstrated lower TOS and OSI levels compared to untreated SAH rats, indicating diazepam’s potential role in counteracting oxidative stress. This outcome complements findings by Sehba et al., who emphasize the importance of managing oxidative stress in SAH to prevent further brain injury^9^. Moreover, Dodd et al. discuss how antioxidants can reduce ROS-mediated damage, providing further context for the antioxidative effects observed in diazepam-treated rats^7^.

The dose of 3 mg/kg diazepam used in this study was selected based on previous rodent studies demonstrating its efficacy in reducing oxidative stress without inducing sedative toxicity. This dose has been shown to modulate oxidative and inflammatory responses effectively while minimizing potential sedative side effects. The choice of this dose is further supported by our findings, where diazepam administration helped reduce oxidative stress markers and inflammatory cytokine levels, highlighting its therapeutic potential in mitigating secondary brain injury following SAH^16^.

Our findings that diazepam treatment may support endogenous antioxidant defenses are notable, especially given the depletion of TAS in untreated SAH rats (Tables 2 and 3). Prior research by Romoli et al. highlights the need for antioxidant interventions in SAH models due to the rapid depletion of natural antioxidants like glutathione and superoxide dismutase following hemorrhage^8^. The maintained TAS levels in the diazepam group suggest that diazepam may help bolster these natural defenses. However, the partial restoration in TAS levels indicates that diazepam alone may not fully prevent oxidative damage, pointing toward a need for combination therapies that could enhance its antioxidative effects, such as vitamins E and C or N-acetylcysteine^6,10^.

### Inflammatory cytokine response and anti-inflammatory effects

The inflammatory cytokine response is another critical component of SAH pathophysiology, contributing to both cerebral vasospasm and delayed ischemic injury. Inflammatory cytokines like IL-1β, IL-6, and TNF-α are elevated following SAH, driving endothelial dysfunction and blood-brain barrier breakdown, which can worsen brain injury^8,19^. Our study revealed significantly higher levels of these cytokines in the SAH group compared to controls (Tables 2 and 3), consistent with research by Alsbrook et al., Dodd et al. and Yamada, H. et al., who reported elevated inflammatory responses in SAH models^5,7,20,21^.

Diazepam-treated rats, showed a reduction in IL-1β and IL-6 levels, indicating an anti-inflammatory effect that aligns with Romoli et al.‘s findings on anti-inflammatory treatments in SAH^8^. This outcome is significant because both IL-1β and IL-6 have been implicated in endothelial dysfunction and increased permeability of the blood-brain barrier^6^. TNF-α, a key driver of neuroinflammation, also decreased in the diazepam group, although it remained elevated compared to controls, suggesting that diazepam may selectively modulate inflammatory pathways rather than fully suppressing immune responses. This selective modulation could benefit patients by reducing harmful inflammation without compromising necessary immune functions, echoing the potential role of anti-TNF therapies discussed by Alsbrook et al.^5,20^.

The observed modulation of HIF-1α further supports diazepam’s potential in modulating hypoxia-driven inflammatory responses. HIF-1α is a key factor in the adaptive response to hypoxia and contributes to inflammatory cascades that worsen ischemic injury^4^. Narotam et al. highlight the role of HIF-1α in exacerbating ischemic injury, suggesting that therapies targeting this pathway could offer additional protection following SAH^4^. Our findings that diazepam reduces HIF-1α levels relative to the untreated SAH group suggest that diazepam could partially alleviate hypoxia-related inflammation (Tables 2 and 3).

### Thiol-disulfide homeostasis

The thiol-disulfide balance, an essential marker of redox status, showed a significant shift towards disulfide formation in the SAH group, suggesting heightened oxidative stress. Elevated disulfide levels can impair vasodilation, contributing to the vasospasm often seen in SAH patients. Total Thiol and Native Thiol levels were notably higher in controls, while Disulfide levels were elevated in the SAH group (Table 3). Diazepam’s partial correction of this imbalance supports its role in stabilizing thiol-disulfide homeostasis, a balance that is crucial for cellular repair processes following injury^10^. This finding aligns with the broader literature indicating that agents restoring thiol-disulfide balance can improve outcomes in oxidative stress-driven diseases^12^.

The ratios of % Native Thiol/Total Thiol and % Disulfide/Total Thiol further confirmed the redox imbalance in the SAH group (Table 3). Diazepam’s moderation of this shift suggests that it may stabilize protein structures and cellular functions, which are compromised by oxidative damage following SAH. As Li et al. discuss, treatments that target redox imbalances hold promise in SAH by potentially preventing vasospasm and improving long-term neurological outcomes^12^.

### Clinical implications and future directions

Our findings suggest that diazepam could serve as an adjunctive treatment in managing secondary injury pathways associated with SAH, focusing on oxidative stress and inflammation. Current SAH treatments largely emphasize maintaining perfusion with Triple H therapy (hypertension, hypervolemia, and hemodilution) and calcium channel blockers like nimodipine^10,11^. However, these strategies do not directly address oxidative and inflammatory damage. Given diazepam’s established safety profile, affordability, and ease of administration, it presents a feasible adjunct to current therapies for SAH.

Future studies could benefit from dose-response analyses to determine whether higher doses of diazepam may yield stronger anti-inflammatory and antioxidant effects without adverse side effects. Additionally, assessing cognitive and functional outcomes following SAH and diazepam treatment would provide insights into its long-term neuroprotective efficacy. The incorporation of additional oxidative stress markers, such as lipid peroxidation and mitochondrial function, could also deepen understanding of diazepam’s effects on oxidative and inflammatory pathways^10,12^.

### Histopathological changes in basilar artery and vasospasm

**Fig. 3:**
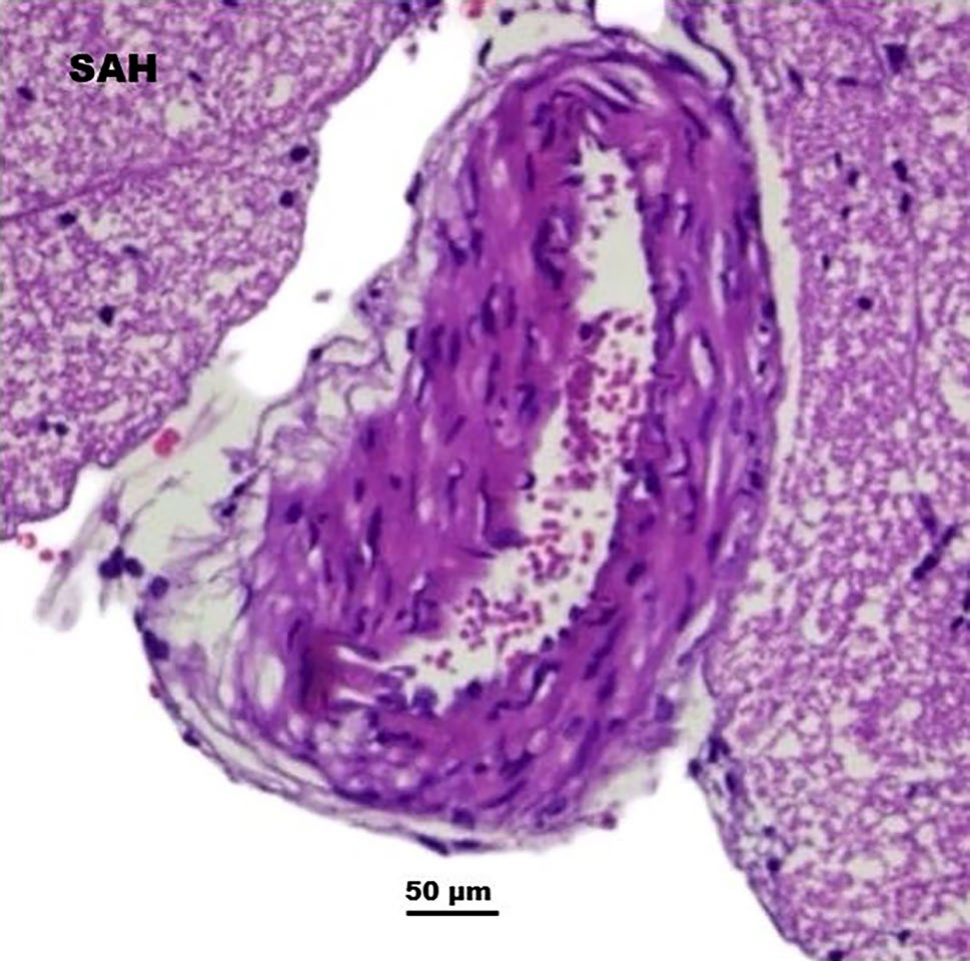
SAH vasospasm and structural alterations in basilar artery following SAH. Histopathological changes in the basilar artery of SAH-induced rats, showing narrowed lumen, increased wall thickness, and presence of inflammatory cells indicative of vasospasm. (Note: Hematoxylin & Eosin staining; magnification, 50x).

**Fig. 4:**
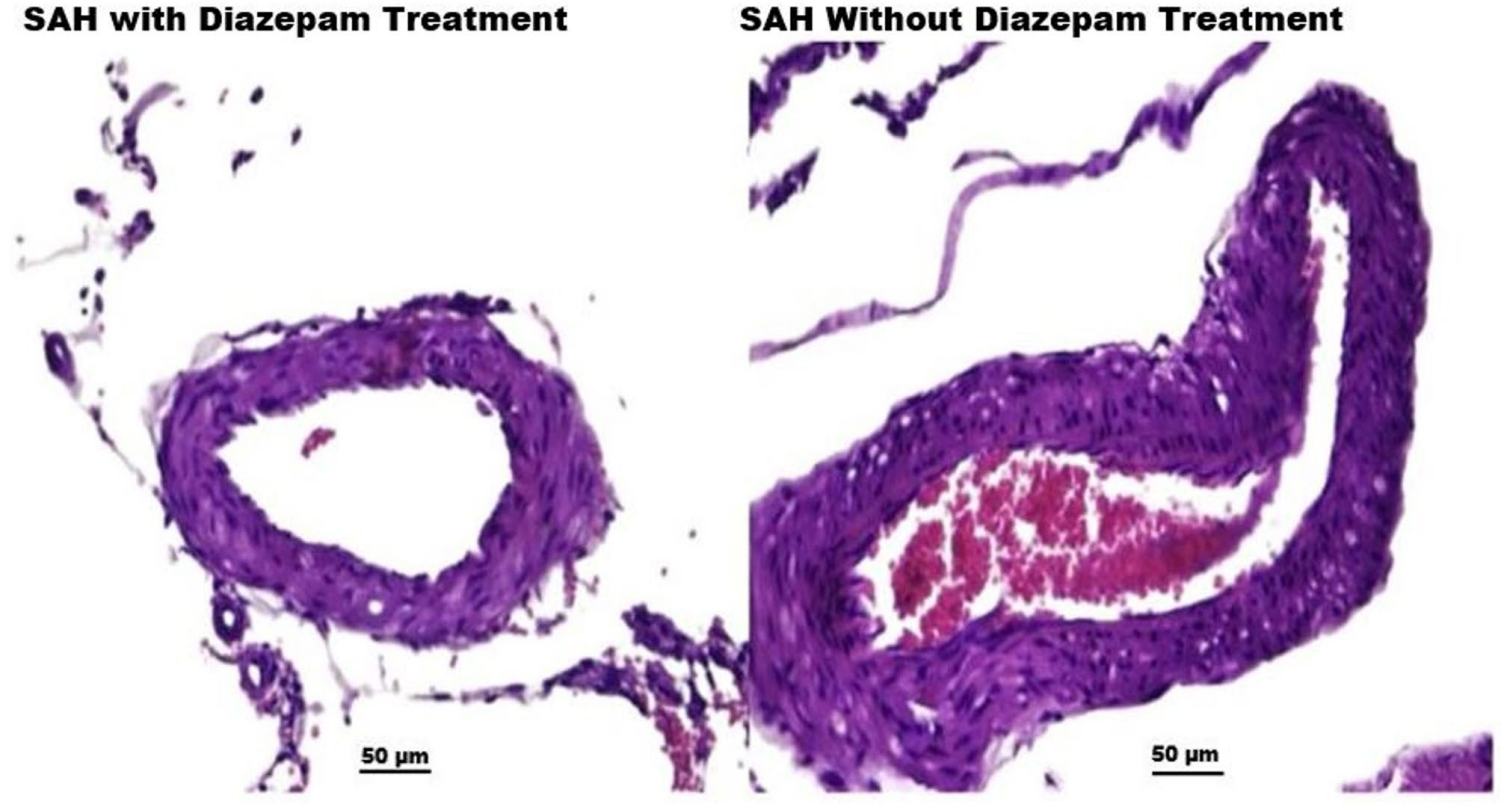
Histological comparison of basilar arteries in sah group with and without diazepam treatment. The image illustrates endothelial cell damage, inflammatory infiltration, and structural disruption in the SAH group (right), while the Diazepam-treated SAH group (left) shows a reduction in vasospasm and inflammation. Wall thickness and lumen diameter suggest a protective effect of diazepam against SAH-induced vascular pathology. (Note: Hematoxylin & Eosin staining; magnification, 50x).

Histopathological analysis of the basilar artery revealed significant vasospasm-related changes in the SAH group, including lumen narrowing, increased wall thickness, and inflammatory infiltration (Figs. 3 and 4). Diazepam treatment mitigated these effects, as observed in the improved basilar artery morphology in the diazepam group (Fig.4).

## Conclusion

In conclusion, this study provides evidence supporting diazepam’s partial protective effects against oxidative stress and inflammatory damage in a rat model of SAH. Diazepam treatment reduced TOS, IL-1β, and IL-6 levels, while partially stabilizing thiol-disulfide homeostasis, suggesting its potential role in mitigating secondary injury following SAH. This study aligns with the broader literature on SAH pathophysiology, which highlights the need for therapies that address oxidative and inflammatory pathways to improve outcomes following SAH^6,8^.

Future research should continue to validate diazepam’s neuroprotective role, ideally through larger preclinical studies and clinical trials. Considering diazepam’s potential in combination with other therapies, such as antioxidants or anti-inflammatory agents, could further enhance its efficacy. Given the complex, multifaceted nature of SAH-induced damage, a comprehensive treatment approach incorporating vasodilation, antioxidant support, and cytokine modulation may yield the best outcomes for SAH patients. Diazepam holds promise as an accessible and effective component of such treatment protocols, potentially improving the clinical management of SAH-related complications and reducing SAH-associated morbidity and mortality. In our study, the effectiveness of diazepam, commonly used for seizure suppression and sedation, in preventing vasospasm has also been demonstrated. Considering the agitation often seen in patients with SAH, administering diazepam may be beneficial both in preventing agitation and in reducing the risk of vasospasm.

## Limitations

This study has several limitations, including a relatively small sample size, which may limit the generalizability of our findings. Furthermore, the study employed a single-dose regimen; thus, additional research is needed to determine the optimal dosing and timing of diazepam administration. Moreover, while this study focused on specific biomarkers, broader analyses incorporating additional oxidative and mitochondrial markers would offer a more comprehensive view of diazepam’s impact on oxidative and inflammatory processes in SAH.

## Data Availability

The datasets used and/or analysed during the current study available from the corresponding author on reasonable request.
