# Correction: Experimental study of the effects of diazepam on vasospasm in a subarachnoid rat model through pathological and biochemical analysis

**DOI:** 10.1038/s41598-025-27976-w

**Published:** 2025-11-18

**Authors:** Eyüp Çetin, Cumaali Demirtaş, Cansu Sönmez, Murat Yücel, Eray Metin Güler, Sarper Kocaoğlu, Hakan Beyaztaş, Emine Demir

**Affiliations:** 1 Neurosurgery Clinic, Haydarpaşa Training and Research Hospital, Health Sciences University, Istanbul, Turkey; 2 Veterinary Clinic, Hamidiye Experimental Animals Laboratory, Health Sciences University, Istanbul, Turkey; 3 Pathology Clinic, Haydarpaşa Numune Hospital, Health Sciences University, Istanbul, Turkey; 4 Faculty of Medicine, Neurosurgery Clinic, Yalova University, Yalova, Turkey; 5 Department of Biochemistry, Hamidiye Medical Faculty, Health Sciences University, Istanbul, Turkey; 6 Department of Medical Biochemistry, University of Health Sciences, Haydarpaşa Numune Health Application and Research Center, Istanbul, Turkey; 7 Neurosurgery Clinic, Training and Research Hospital, Istanbul, Turkey; 8 Department of Medical Biochemistry, Hamidiye Institute of Health Sciences, University of Health Sciences Turkey, Istanbul, Turkey; 9 Department of Neurosurgery, Üsküdar State Hospital, Istanbul, Turkey

Correction to: *Scientific Reports* 10.1038/s41598-025-08325-3, published online 09 August 2025

The original version of this Article contained an error in the spelling of the author Sarper Kocaoğlu which was incorrectly given as Sarper Kocoğlu.

In addition, Eray Metin Güler was incorrectly affiliated with ‘Department of Medical Biochemistry, Hamidiye Institute of Health Sciences, University of Health Sciences Turkey, Istanbul, Turkey’. The correct affiliations are ‘Department of Biochemistry, Hamidiye Medical Faculty, Health Sciences University, Istanbul, Turkey’ and ‘Department of Medical Biochemistry, University of Health Sciences, Haydarpaşa Numune Health Application and Research Center, Istanbul, Turkey’.

Furthermore, Hakan Beyaztaş was incorrectly affiliated with ‘Department of Medical Biochemistry, University of Health Sciences, Haydarpaşa Numune Health Application and Research Center, Istanbul, Turkey’. The correct affiliations are ‘Department of Biochemistry, Hamidiye Medical Faculty, Health Sciences University, Istanbul, Turkey’ and ‘Department of Medical Biochemistry, Hamidiye Institute of Health Sciences, University of Health Sciences Turkey, Istanbul, Turkey’.

The original Article has been corrected.

